# Frequency, Clinical Characteristics, Anatomical Distribution, and Outcomes of Embolic Complications of Cardiac Myxoma in Contemporary Cohorts: Protocol for a Systematic Review and Meta-Analysis

**DOI:** 10.2196/92926

**Published:** 2026-06-24

**Authors:** Arun Dahil, Callum Hill, Cameron Pinn, David Hardisty, Piotr Wasilewski, Matthew Turner, Michaela Scheuermann-Freestone

**Affiliations:** 1 Faculty of Medicine, University of Southampton, University Rd, Southampton, Southampton, England, SO17 1, United Kingdom, 44 7415015896; 2 Department of Cardiology, Basingstoke and North Hampshire Hospital, Basingstoke, England, United Kingdom

**Keywords:** cardiac myxoma, embolism, cerebral infarction, stroke, systematic review

## Abstract

**Background:**

Cardiac myxomas (CMs) are the commonest benign primary cardiac tumors, most frequently originating from the left atrium and occasionally from the right atrium. Despite being histologically benign, CMs can cause myriad serious embolic complications, including stroke, acute coronary syndrome, limb ischemia, and visceral infarction. While previous studies have explored risk factors for embolization, there is a lack of papers comprehensively summarizing the frequency, anatomical distribution, clinical patterns, and management of CM-related embolization.

**Objective:**

This review aims to provide a comprehensive synthesis of embolic complications associated with CMs, highlighting patterns, management strategies, and gaps in the current literature.

**Methods:**

A systematic review will be conducted in accordance with the PRISMA-P (Preferred Reporting Items for Systematic Reviews and Meta-Analyses Protocols) guidelines. MEDLINE, Embase, Scopus, CINAHL, the Cochrane Library, and PubMed will be searched for studies reporting embolic complications in patients with histologically or radiologically confirmed CMs. Eligible study designs include large case series, cohort studies, and registries. Six reviewers will independently perform title and abstract and full-text screening in pairs across 3 screening groups, with disagreements resolved through discussion and senior reviewer adjudication where necessary. Data will be extracted by 3 reviewers, with the extracted data independently verified for accuracy. Discrepancies will be resolved through discussion or third-party adjudication. Risk of bias will be assessed using the Joanna Briggs Institute tools.

**Results:**

The review will summarize reported frequencies and anatomical distribution of embolic events, clinical presentations, associations with tumor characteristics, and management strategies. Title and abstract screening were completed in early April 2026, and full-text screening commenced in late April 2026. Data extraction and synthesis were completed in May 2026. We anticipate publication of the findings in September 2026.

**Conclusions:**

This review aims to provide a comprehensive synthesis of embolic complications associated with CMs, highlighting patterns, management strategies, and gaps in the current literature. The findings aim to improve clinical recognition, inform clinical management, and guide future research.

## Introduction

### Background

Cardiac myxomas (CMs) are the most common benign primary cardiac tumor, with most originating from the left atrium at either the mitral annulus or fossa ovalis of the interatrial septum, although some myxomas may also be seen in the right atrium [1]. CMs are very rare, with an estimated prevalence of 0.02% in the United Kingdom [2]. CMs are thought to arise from multipotent mesenchymal cells capable of differentiating along multiple lineages, including neural and endothelial, as evidenced by their expression of corresponding immunohistochemical markers [3]. Most CMs occur sporadically, while a small percentage exhibit a familial association and can contribute to genetic syndromes such as Carney complex [4]. Many CMs are associated with a mutation in the PRKAR1A gene, which encodes a regulatory subunit of protein kinase A. Dysfunction in protein kinase A regulation leads to uncontrolled cellular proliferation [4].

Patients can present asymptomatically or with clinical features that can be classified as cardiac, constitutional, embolic, or obstructive [1]. Cardiac symptoms may include dyspnea, palpitations, and syncope, while obstructive symptoms are characterized by mitral regurgitation and tricuspid stenosis, leading to clinical consequences such as pulmonary edema, positional syncope, and Budd-Chiari syndrome. Constitutional symptoms such as fatigue, fever, and weight loss may result from CMs that produce interleukin-6 and vascular endothelial growth factor [5]. Finally, emboli may arise from the separation of CM fragments or the formation and separation of tumor surface thrombi and can cause ischemic cerebral infarction as well as pulmonary embolization [6]. CMs are primarily diagnosed through transthoracic and transesophageal echocardiography and can be analyzed via histopathology after resection [7,8]. The embolic potential of CMs can be derived from the morphological characteristics of the tumor, such as regularity of shape, surface texture, and attachment to the underlying tissue [9].

Embolic complications represent some of the most severe manifestations of CMs, yet research remains largely confined to isolated case reports and retrospective single-center case series. Published case reports have described a broad spectrum of embolic manifestations, including ischemic stroke, brain metastases, retinal artery occlusion, acute limb ischemia, and coronary artery embolization [10-14]. These reports highlight the heterogeneous clinical presentation of CM-related embolization but are limited by inconsistent reporting standards, variable follow-up, and the absence of standardized outcome measures.

Previous systematic reviews such as those by Liu et al [15] and Qureshi et al [16] have used meta-analytic approaches to identify embolic risk factors, including hypertension, tumor mobility, and surface characteristics. Furthermore, Lacerda Teixeira et al [17] used clinical and echocardiographic data to investigate predictors of embolization within a patient cohort. While these studies focused on risk factors, systematic exploration of the resulting embolic outcomes remains limited. Oktaviono et al [18] and Rabiee Rad et al [19] conducted systematic reviews on the general clinical characteristics, tests, and surgical results of CMs, but each addressed embolization only briefly, with Oktaviono et al [18] discussing mechanisms rather than clinical sequelae. Similarly, Saputra et al [20] characterized the clinical manifestations and management of pulmonary valve myxomas without discussing embolic consequences. In contrast, Bandlamuri et al [21] examined cerebral embolic complications specifically within pediatric populations and focused on neurosurgical management.

Although existing systematic reviews address aspects of CMs and embolization, none have comprehensively summarized the frequency, anatomical distribution, clinical presentation, and management strategies across the full spectrum of embolic events. This gap in the literature limits clinical guidance on recognition and urgency of intervention, motivating this review.

### Primary Review Question

The primary review question is as follows: among adults with sporadic CM, what is the frequency and anatomical distribution of myxoma-related embolic complications reported in contemporary clinical studies? A population, intervention, comparator, and outcome criteria for the primary review question is shown in Table 1.

**Table 1.** Population, intervention, comparator, and outcome (PICO) criteria for the primary review question.

PICO element	Criteria
Population	Adults aged ≥18 years with sporadic CM^a^ confirmed via histology, imaging, operative findings, or clear study author diagnosis
Intervention	CM with embolic complications classified as definite or probable myxoma-related embolism
Comparator	Adults with CM without embolic complications where reported; no comparator required for descriptive frequency estimates
Outcomes	Primary: frequency and anatomical distribution of embolic complications; secondary: clinical presentations, management strategies, perioperative outcomes, recurrence, mortality, and associations with tumor characteristics

a CM: cardiac myxoma.

Embolization is described as the fragmentation of the CM itself or the formation and dispersal of surface thrombi on the heart tumor.

### Secondary Review Questions

In addition to the primary review question, this systematic review will address the following secondary questions:

1. What are the clinical presentations (eg, stroke phenotypes, pulmonary infarction, acute coronary syndrome, and limb ischemia) and management strategies of CM-related embolic complications?
2. What are the reported outcomes following embolic complications (mortality, tumor recurrence, neurological deficit, and perioperative details)?

## Methods

### Protocol, Registration, and Amendments

This protocol has been developed in line with the PRISMA-P (Preferred Reporting Items for Systematic Reviews and Meta-Analyses Protocols) guidance [22]. The systematic review protocol is registered in PROSPERO (CRD420261299634). A systematic review was selected over a scoping review as the primary objective involves the calculation of frequency estimates and, where data permit, pooled effect measures via meta-analysis. These require the methodological rigor of a systematic review as opposed to broader descriptive mapping. Any amendments to or deviations from this protocol will be documented and presented as supplementary information alongside the final manuscript. Reasoning will be given for any deviation.

### Eligibility Criteria

#### Participants

This review will include studies involving adult participants aged 18 years or older diagnosed with sporadic CM in whom at least one embolic complication attributable to the tumor has been reported. Studies including mixed adult and pediatric populations will be included as many studies do not stratify adult and pediatric cohorts, and the proportion of pediatric patients is often small or unreported. Excluding mixed-age cohorts would risk the omission of valuable data relevant to this relatively rare condition. Where available, adult-specific data will be extracted separately. Patients whose embolic events occurred either before diagnosis or during the clinical course of CM are eligible for inclusion. Studies exclusively focusing on patients with hereditary CM (Carney complex) will be excluded.

Participants may originate from various clinical settings, including outpatient evaluation, emergency presentations, or inpatient hospital admissions. Studies exclusively involving cardiac tumors other than myxoma will be excluded unless data specific to CM can be separately extracted. No restrictions will be applied based on sex, geographic region, or clinical comorbidities.

#### Exposure and Comparators

Eligible studies must report the presence of diagnosed CM associated with one or more embolic events. The threshold for attribution is the definite or probable expression of causation of embolism due to a CM in the relevant study. Attribution categories are defined in Table 2.

**Table 2.** Threshold attribution criteria of thromboembolic events due to cardiac myxoma^a^.

Attribution category	Required criteria	Use in synthesis
Definite	Confirmed cardiac myxoma plus pathological, imaging, operative, or retrieved embolus evidence linking the embolus to the myxoma	Included in primary analysis
Probable	Confirmed cardiac myxoma, temporally related embolic event before diagnosis or resection, anatomically plausible embolic distribution, and no stronger alternative embolic source reported	Included in primary analysis
Possible	Confirmed cardiac myxoma and embolic event, but limited reporting of competing causes or diagnostic workup	Excluded from primary analysis
Unrelated or indeterminate	Alternative embolic source identified, implausible anatomy, unclear timing, or insufficient information	Excluded from primary analysis

a Comparators are not required for study inclusion. Studies comparing groups with embolic and nonembolic complications will be included if data for each group are available. Data comparing embolic vs nonembolic presentations or different management approaches will be extracted when reported.

#### Outcomes

The primary outcomes of interest include the frequency and anatomical distribution of embolic complications associated with CM. Eligible embolic events included the following: (1) cerebral embolism or ischemic stroke; (2) coronary embolism or myocardial infarction; (3) peripheral arterial embolism involving limbs; (4) visceral embolism involving organs such as the kidneys, spleen, or mesenteric circulation; (5) pulmonary embolic events where relevant; (6) and multiple or systemic embolic events. Septic embolization and embolization attributable to a source other than the CM were excluded.

Secondary outcomes will include, where available, clinical presentation of CM-related embolization, tumor location (atrial or ventricular), tumor size, tumor morphology, management strategies (surgical or medical treatment), patient follow-up, and clinical outcomes (neurological deficit, recurrence, perioperative information, and mortality).

#### Types of Studies

This review will include primary study designs reporting embolic complications associated with CM, including observational cohort studies (prospective or retrospective), randomized controlled trials, case-control studies, cross-sectional studies, and case series with 10 or more patients. To improve data reliability and reduce bias associated with single-case and small series reports, only studies including 10 or more patients will be included. This approach prioritizes more robust observational evidence while maintaining consistency across included studies. Included studies must discuss embolization in patients with CM, with embolic complications described in the abstract. Larger case series published before the year 2000 will be excluded to improve contemporary relevance. Narrative or systematic reviews, editorials, commentaries, and opinion pieces without primary data; conference abstracts that have not undergone peer review; and laboratory studies will be excluded. If a study abstract is not accessible, the study will be excluded from extraction. Studies describing septic embolization or emboli from a source other than CM will be excluded. Only English-language studies or studies with an available English translation will be included due to feasibility constraints, with no time or geographic restrictions.

### Information Sources

The search will be conducted using the following bibliographic databases through the online library portal of the University of Southampton: MEDLINE (Ovid), Embase (Ovid), PubMed, Scopus, CINAHL, and the Cochrane Library. Gray literature sources, including ClinicalTrials.gov and the World Health Organization International Clinical Trials Registry Platform, will also be reviewed to identify relevant unpublished or difficult-to-locate material. The inclusion of these databases will provide comprehensive coverage of information about the embolic complications of CMs.

### Search Strategy

A structured search strategy will be carried out to identify the relevant literature assessing the characteristics of embolic complications in patients with CMs. We will use a combination of keywords and controlled vocabulary terms to maximize coverage. Search terms will be organized into 2 categories: one related to cardiac anatomy and pathology (*heart*, *cardiac*, and *myxoma*) and the other related to *embolism*. Searches will be conducted from database inception to the date of the final search without date restrictions. In addition to database searching, backward citation searching of reference lists of the included studies and relevant reviews will be performed. Forward citation searching will also be conducted using Google Scholar, Scopus, or Web of Science, where available. Any studies identified through forward or backward citation screening will undergo the same screening and be subject to the same eligibility criteria as studies identified via database searches. Full search strategies are available in Multimedia Appendix 1.

### Data Collection and Selection Process

The selection process will follow a standardized approach comprising title screening, abstract review, and full-text analysis. Six reviewers (AD, CH, CP, PW, DH, and MT) will independently perform title and abstract and full-text screening in pairs across 3 screening groups, with disagreements resolved through discussion and senior reviewer (AD) adjudication where necessary. Records will be classified as “include,” “exclude,” or “uncertain.” Reasons for exclusion at this stage will be documented. Data will be extracted into a standardized data extraction form using a CSV format by 3 reviewers (AD, CP, and DH), with each reviewer’s work independently verified by the other reviewers.

### Data Management

Search results from all included databases will be imported into Rayyan (Qatar Computing Research Institute) for deduplication and screening. When multiple publications describe overlapping patient populations, the report containing the most complete dataset will be included to avoid duplication. Any discrepancies between data extraction and verification will be resolved through discussion, with additional reviewer (CH) adjudication where consensus cannot be reached. Before full extraction, the data extraction form will be piloted on a subset of studies and refined to ensure consistency between reviewers. A predefined set of outcomes will be extracted to comprehensively explore embolic complications of CMs.

The characteristics of each included study will be described, including first author, year of publication, country, study design, and sample size. Population characteristics will also be recorded, including participant age and sex. Potential confounding variables such as smoking status, alcohol use, and preexisting comorbidities will be extracted where reported. Tumor-specific variables such as location and size will be documented, along with details of embolic events, including type, anatomical site, and clinical presentation. Clinical presentations of embolic complications will be extracted where reported, including ischemic stroke or transient ischemic attack, acute coronary syndrome or myocardial infarction, acute limb ischemia, renal infarction, splenic infarction, mesenteric ischemia, pulmonary embolism, pulmonary infarction, syncope, focal neurological deficit, dyspnea, and limb pain or pallor. Diagnostic modalities used to identify CMs and embolization (eg, transthoracic echocardiography, transesophageal echocardiography, computed tomography, magnetic resonance imaging, and angiography) will be recorded. Perioperative outcomes will also be extracted where reported, including timing of surgery after embolic presentation, use of cardiopulmonary bypass, perioperative stroke, bleeding, recurrent embolism, postoperative neurological status, and intensive care admission. Finally, management strategies and outcomes related to CM embolization will be extracted, including surgical intervention, thrombectomy, anticoagulation, neurological recovery, mortality, and recurrence.

### Data Synthesis

Findings from the included studies will be synthesized using a structured approach to accommodate anticipated heterogeneity in study design, patient populations, and outcome reporting. As many included studies are expected to be case series providing descriptive clinical data, synthesis will involve both systematic summarization of reported findings and quantitative pooling.

Data will first be organized into a structured data table summarizing study characteristics, patient demographics, tumor features, embolic manifestations, management strategies, and clinical outcomes. Embolic complications will be grouped into anatomical categories to enable comparison between studies. Tumor characteristics, such as location, morphology, and size, will also be recorded. We will report summary-level metrics, including the location of embolic events, outcome, and management strategy. We aim to analyze patterns across reported characteristics, with attention paid to recurrent clinical features and factors potentially associated with embolic risk.

Where comparative data are available, associations between tumor characteristics and embolic complications will be explored. For categorical variables, odds ratios or risk ratios with 95% CIs will be extracted or calculated where possible. For continuous variables, mean or median differences will be extracted where reported.

Where at least 3 studies report comparable denominator data for CM populations and comparable definitions of embolic complications, pooled frequency estimates will be calculated using random-effects meta-analysis, with results reported with 95% CIs and heterogeneity assessed using the *I*^2^ statistic. Sensitivity analyses will be conducted where feasible to explore the influence of study characteristics or risk of bias on pooled results. Where substantial methodological heterogeneity is present and quantitative synthesis is not appropriate, results will be summarized narratively and descriptively rather than pooled quantitatively.

### Risk-of-Bias Assessment

Risk of bias will be assessed using the Joanna Briggs Institute critical appraisal tools, with study design–specific checklists applied according to the methodological design of each included study [23]. Cohort studies, case series, and case reports will be appraised using their respective Joanna Briggs Institute tools. Two reviewers will independently assess each study, with discrepancies resolved through discussion or consultation with a third reviewer where necessary.

Before full assessment, reviewers will calibrate a subset of studies to ensure consistent interpretation and application of appraisal criteria. Risk-of-bias judgments will be summarized in tabular form and considered during the interpretation of study findings. Study design and risk of bias will not be used to exclude studies but will be taken into consideration when interpreting patterns and conclusions from the data.

### Meta-Biases

Publication bias is anticipated given the selective and preferential reporting of rare embolic events or presentations associated with CM, particularly in case reports or case series. This may result in overrepresentation of severe outcomes and underreporting of asymptomatic or less severe cases. Where quantitative synthesis is performed, we will explore the effects of publication bias or small sample sizes on any potential conclusions.

### Confidence in Cumulative Evidence

The overall certainty of evidence is expected to be limited due to the significant number of case reports and observational studies with small sample sizes. A limited number of larger cohort studies may provide evidence of higher certainty. Where a sufficient number of cohort studies are used, the GRADE (Grading of Recommendations Assessment, Development, and Evaluation) framework will be used to assess evidence certainty [24].

### Ethical Considerations

This study involves secondary analysis of published data and, therefore, does not require formal research ethics approval. The review will be conducted by a multidisciplinary team with experience in cardiology and quantitative analysis. All authors are affiliated with the University of Southampton.

### Dissemination

The findings will be disseminated through publication in a peer-reviewed journal. The results may also be disseminated in the form of conference abstracts or presentations. Where feasible, anonymized extracted data and analysis code (eg, for meta-analyses) will be made available in an open repository.

## Results

**Figure 1.**
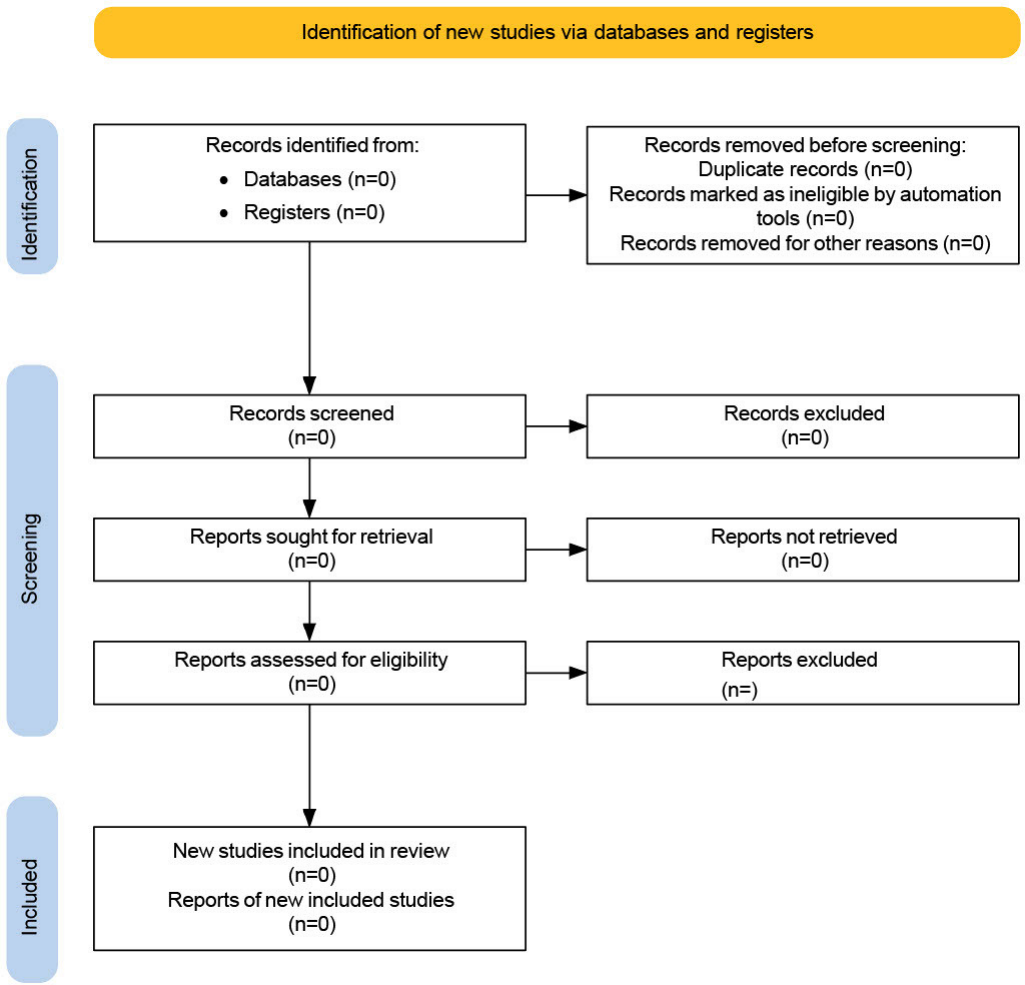
PRISMA (Preferred Reporting Items for Systematic Reviews and Meta-Analyses) study selection and screening flowchart.

In February 2026, a preliminary scoping search was conducted to assess the types of studies available and assess whether any previous systematic reviews were registered or existed in the current literature that answered our research questions. The database searches were last run in February 2026, and all included studies were retrieved as of this date. Title and abstract screening were completed in early April 2026, and full-text screening commenced in late April 2026. Data extraction and synthesis were completed in May 2026, with a final draft of the manuscript to be finalized by September 2026. A flow diagram following PRISMA (Preferred Reporting Items for Systematic Reviews and Meta-Analyses) guidelines will be used to record the data selection process (Figure 1). This study did not receive any specific funding from any agency in the public, commercial, or not-for-profit sectors.

The results of this review will be presented using structured summary tables describing study characteristics, including authorship, year of publication, study design, patient demographics, tumor features, reported embolic complications (including anatomical distribution and clinical presentation), diagnostic approaches, and management strategies.

## Discussion

### Expected Principal Findings

This systematic review is expected to quantify the overall frequency of embolization among CM cohorts. This review also aims to show that embolic complications of CMs are clinically heterogeneous, with cerebral embolism likely to be the most frequently reported presentation followed by peripheral, visceral, coronary, pulmonary, or multi-territory embolic events. We anticipate that the anatomical distribution of embolic risk will vary according to covariates, including tumor morphology, location, and patient comorbidities, although the strength of these associations and the quality of evidence generated may be limited by study heterogeneity. Clinical presentation will vary, with many patients potentially presenting with strokelike symptoms, acute limb ischemia, acute coronary syndrome, and pulmonary emboli. It is predicted that tumor recurrence will affect a small subset of cohorts, as will mortality. Many patients may experience longer-term outcomes such as permanent neurological damage, limb amputation, and vision loss. We anticipate that the most common diagnostic modality used will be echocardiography, followed by histopathology.

We expect to identify variation in acute management strategies, including surgical excision, thrombectomy, anticoagulation, and supportive management, with variable reporting of longer-term outcomes.

### Comparison to Prior Work

Previous literature on embolic complications of CMs has largely comprised narrative reviews, retrospective single-center case series, cohort studies, and case reports. Existing studies have often been limited by small sample sizes; selective focus on specific embolic territories; and a lack of contemporary synthesis across clinical presentations, management strategies, and outcomes. For example, Lee et al [25] evaluated central nervous system manifestations of CM in a retrospective cohort study, demonstrating the predominance of cerebral infarction in CM-related embolization. Although broader reviews have acknowledged embolization as part of the general clinical presentation of CM, they have not systematically summarized embolic complications across anatomical territories or outcomes [26]. Much of the available literature exists in the form of isolated case reports describing a wide range of embolic phenomena, including cerebral infarction, peripheral vascular embolization, retinal embolization, and visceral embolization [27-30]. Larger retrospective single-center series such as that by Amemiya et al [9] have attempted to capture multi-territory embolic outcomes, reporting cerebral, coronary, renal, and peripheral limb events. However, these studies remain constrained by small cohorts and retrospective designs. Overall, the literature is heterogeneous in reporting standards, management approaches, and outcome assessment.

Therefore, this systematic review aims to provide a comprehensive and contemporary synthesis of embolic complications associated with CM, including their frequency, anatomical distribution, clinical presentation, management, and outcomes across published studies.

### Strengths and Limitations

This systematic review encompasses 6 major databases, with supplementation from gray literature sources. The breadth of collected data minimizes the risk of studies being missed. Both backward and forward citation searching may capture literature that does not appear in routine database searches. Our approach includes comprehensive eligibility criteria that capture the full spectrum of complications associated with CMs across multiple study designs. The inclusion of tiered attribution criteria that classify embolic events as definite, probable, possible, or unrelated (Table 2) creates a framework to address the challenge of determining CM-related embolization as opposed to the sole reliance on the clinical judgment of the original authors. Our framework increases the internal validity of frequency and distribution estimates of CM-related embolization.

Six independent reviewers will conduct title and abstract screening in duplicate across 3 groups, with structured adjudication for disagreements. This creates a robust, replicable methodology for screening. This review prioritizes observational evidence with larger sample sizes by restricting the inclusion of case series to 10 or more patients and excluding case reports. This threshold avoids the distortion that highly selected single-case publications introduce into frequency calculations, where rare or dramatic presentations are disproportionately represented, while maintaining a breadth of coverage appropriate to a rare condition.

Anticipated heterogeneity in study design, reporting standards, and outcome definitions has been explicitly acknowledged and addressed through a tiered, prespecified synthesis strategy. Descriptive tabulation will be applied to all included studies. Narrative synthesis will be used where quantitative pooling is inappropriate, and random-effects meta-analysis will be conducted where studies provide comparable denominator data and concordant outcome definitions. This avoids the inappropriate pooling of heterogeneous data while remaining statistically rigorous where the evidence permits.

Despite the many strengths of our review, we address limitations such as the exclusion of case series with fewer than 10 patients and individual case reports, which constitute a substantial portion of the published literature. This choice was made to improve the reliability of frequency estimates and reduce distortion from single-case publications. However, this carries the risk of excluding rare forms of embolization. Furthermore, the available evidence is expected to be heterogeneous, derived from small observational studies and case series, which are inherently susceptible to selection bias, incomplete reporting, and variable methodological quality. Variability in how embolization is defined and attributed across studies will limit direct quantitative comparison and may reduce the applicability of pooled estimates. This limitation is partially mitigated by the attribution criteria, anatomical categorization, and a tiered synthesis strategy that defaults to narrative synthesis where quantitative pooling is methodologically inappropriate. Nevertheless, residual heterogeneity is likely to constrain the certainty of the conclusions drawn from any pooled analysis.

Embolic complications are more likely to be published when the clinical presentation is dramatic or the outcome is severe and less likely to be published when the clinical course is uncomplicated or silent. This selective reporting could inflate observed frequencies of severe manifestations of embolization. To mitigate this, pooled frequency estimates will be restricted to studies with clearly defined denominators representing all patients with CM rather than only those with embolic events. Large case series published before the year 2000 are excluded due to evolving diagnostic modalities and clinical description of CM, improving the contemporary application of our findings. While this improves relevance to current clinical practice, it risks excluding historically significant large series that may contribute important data on embolic frequency, anatomical distribution, and long-term outcomes.

### Future Directions

While our systematic review summarizes frequency, distribution, and outcomes of CM-related embolization, several important gaps are highlighted in the current literature, warranting future investigation. First, future studies should adopt standardized embolic workup protocols including the systematic exclusion of competing sources of embolization (atrial fibrillation and atherosclerosis); histopathological examination of surgically removed embolic material; and the formal classification of embolic events as definite, probable, or possible myxoma related. Furthermore, the heterogeneous nature of the existing literature may mean that outcomes could vary between cohorts. Therefore, standardized outcome reporting, such as the National Institutes of Health Stroke Scale and modified Rankin Scale, could mean that clinical outcomes could be meaningfully compared across studies. Preliminary scoping of the literature reveals many retrospective studies operating within single centers, which are subject to limited sample sizes. A prospective multicenter registry would provide larger sample sizes for meaningful statistical analysis, standardized data collection protocols, and generalizable findings across different health care settings and populations. Following our review, the development of a clinical risk score for embolism in patients with CM would be of significant clinical value. Variables identified in existing literature include tumor morphology, size, left atrial diameter, atrial fibrillation, and platelet parameters. A validated risk stratification tool could guide surgical urgency decisions.

### Conclusions

This systematic review will provide a comprehensive summary of the frequency, anatomical distribution, clinical manifestations, and management implications of embolic complications of CM. Through a structured, replicable methodology, relevant studies will be captured, followed by a combined qualitative-quantitative analysis of the data. Our findings may provide a structured framework that could aid in future clinical management.

## Supplementary material

10.2196/92926 Multimedia Appendix 1 Complete set of search strategies.

10.2196/92926 Checklist 1 PRISMA-P checklist.

## Abbreviations

CM: cardiac myxoma
GRADE: Grading of Recommendations Assessment, Development, and Evaluation
PRISMA: Preferred Reporting Items for Systematic Reviews and Meta-Analyses
PRISMA-P: Preferred Reporting Items for Systematic Reviews and Meta-Analyses Protocols
