# Supplementary material for: Frequency, Clinical Characteristics, Anatomical Distribution, and Outcomes of Embolic Complications of Cardiac Myxoma in Contemporary Cohorts: Protocol for a Systematic Review and Meta-Analysis
Source: JMIR Res Protoc. 2026 Jun 24;15:e92926. doi: 10.2196/92926 (PMC13293564; doi:10.2196/92926)
Supplement: Multimedia Appendix 1 [file resprot-v15-e92926-s001.docx]

**Table 1. A summary of the included databases, their respective search strategies and the results from each search**

| **Database** | **Search strategy** | **Results** |
| --- | --- | --- |
| Scopus | TITLE-ABS-KEY ( ( "cardiac myxoma" OR "heart myxoma" OR "atrial myxoma" OR "left atrial myxoma" OR "right atrial myxoma" OR "ventricular myxoma" OR "intracardiac myxoma" OR "intracavitary myxoma" ) AND ( embol* OR thromboemboli* OR thromboembol* OR "ischaemic stroke" OR "ischemic stroke" OR "cerebral infarct*" OR "cerebral embol*" OR "coronary embol*" OR "myocardial infarct*" OR "peripheral embol*" OR "arterial embol*" OR "limb ischaemi*" OR "limb ischemi*" OR "visceral embol*" OR "renal infarct*" OR "splenic infarct*" OR "mesenteric embol*" OR "pulmonary embol*" OR "systemic embol*" OR "tumour embol*" OR "tumor embol*" OR "myxoma fragment*" )) | 1959 |
| Cochrane | #1 "cardiac myxoma" OR "heart myxoma" OR "atrial myxoma"  OR "left atrial myxoma" OR "right atrial myxoma"  OR "ventricular myxoma" OR "intracardiac myxoma"  #2 embol* OR thromboemboli* OR thromboembol*  OR "ischaemic stroke" OR "ischemic stroke"  OR (cerebral NEXT infarct*)  OR (cerebral NEXT embol*)  OR (coronary NEXT embol*)  OR (peripheral NEXT embol*)  OR (arterial NEXT embol*)  OR (limb NEXT ischaemi*)  OR (limb NEXT ischemi*)  OR (visceral NEXT embol*)  OR (renal NEXT infarct*)  OR (splenic NEXT infarct*)  OR (pulmonary NEXT embol*)  OR (systemic NEXT embol*)  OR (tumour NEXT embol*)  OR (tumor NEXT embol*)  OR (myxoma NEXT fragment*)  #3 #1 AND #2 | 1 |
| CINAHL | #1 TI ( "cardiac myxoma" OR "heart myxoma" OR "atrial myxoma"  OR "left atrial myxoma" OR "right atrial myxoma"  OR "ventricular myxoma" OR "intracardiac myxoma"  OR "intracavitary myxoma" )  OR AB ( "cardiac myxoma" OR "heart myxoma" OR "atrial myxoma"  OR "left atrial myxoma" OR "right atrial myxoma"  OR "ventricular myxoma" OR "intracardiac myxoma"  OR "intracavitary myxoma" )  #2 TI ( embol* OR thromboemboli* OR thromboembol*  OR "ischaemic stroke" OR "ischemic stroke"  OR "cerebral infarct*" OR "cerebral embol*"  OR "coronary embol*" OR "peripheral embol*"  OR "arterial embol*" OR "limb ischaemi*"  OR "limb ischemi*" OR "visceral embol*"  OR "renal infarct*" OR "splenic infarct*"  OR "mesenteric embol*" OR "pulmonary embol*"  OR "systemic embol*" OR "tumour embol*"  OR "tumor embol*" OR "myxoma fragment*" )  OR AB ( embol* OR thromboemboli* OR thromboembol*  OR "ischaemic stroke" OR "ischemic stroke"  OR "cerebral infarct*" OR "cerebral embol*"  OR "coronary embol*" OR "peripheral embol*"  OR "arterial embol*" OR "limb ischaemi*"  OR "limb ischemi*" OR "visceral embol*"  OR "renal infarct*" OR "splenic infarct*"  OR "mesenteric embol*" OR "pulmonary embol*"  OR "systemic embol*" OR "tumour embol*"  OR "tumor embol*" OR "myxoma fragment*" )  #3 #1 AND #2 | 208 |
| Medline (Ovid) | 1. Myxoma/  2. cardiac myxoma.ti,ab.  3. atrial myxoma.ti,ab.  4. (myxoma adj3 (cardiac or atrial or heart)).ti,ab.  5. 1 OR 2 OR 3 OR 4  6. Embolism/  7. Thromboembolism/  8. Stroke/  9. Ischemic Attack, Transient/  10. embol*.ti,ab.  11. thromboembol*.ti,ab.  12. (stroke OR TIA OR cerebral infarct*).ti,ab.  13. (coronary embol* OR myocardial infarct* OR acute coronary syndrome).ti,ab.  14. (limb isch?em* OR peripheral embol*).ti,ab.  15. (renal infarct* OR splenic infarct* OR mesenteric isch?em* OR visceral infarct*).ti,ab.  16. retinal artery occlusion.ti,ab.  17. pulmonary embol*.ti,ab.  18. 6 OR 7 OR 8 OR 9 OR 10 OR 11 OR 12 OR 13 OR 14 OR 15 OR 16 OR 17  19. 5 AND 18 | 1400 |
| Embaase (Ovid) | 1.cardiac myxoma/  2. cardiac myxoma.ti,ab.  3. atrial myxoma.ti,ab.  4. (myxoma adj3 (cardiac or atrial or heart)).ti,ab.  5. 1 or 2 or 3 or 4    6. embolism/  7. thromboembolism/  8. stroke/  9. transient ischemic attack/  10. embol*.ti,ab.  11. thromboembol*.ti,ab.  12. (stroke or TIA or cerebral infarct*).ti,ab.  13. (coronary embol* or myocardial infarct* or acute coronary syndrome).ti,ab.  14. (limb isch?em* or peripheral embol*).ti,ab.  15. (renal infarct* or splenic infarct* or mesenteric isch?em* or visceral infarct*).ti,ab.  16. retinal artery occlusion.ti,ab.  17. pulmonary embol*.ti,ab.  18. 6 or 7 or 8 or 9 or 10 or 11 or 12 or 13 or 14 or 15 or 16 or 17  19. 5 and 18 | 2390 |
| PubMed | ("Myxoma"[Mesh] OR"cardiac myxoma"[Title/Abstract] OR "atrial myxoma [Title/Abstract] OR (myxoma[Title/Abstract] AND (cardiac[Title/Abstract] OR atrial[Title/Abstract] OR heart[Title/Abstract]))) AND ("Embolism"[Mesh] OR "Thromboembolism"[Mesh] OR "Stroke"[Mesh] OR "Ischemic Attack, Transient"[Mesh] OR embol [Title/Abstract] OR thromboembol*[Title/Abstract] OR stroke[Title/Abstract] OR TIA[Title/Abstract] OR "cerebral infarct” [Title/Abstract] OR "coronary embol*"[Title/Abstract] OR "myocardial infarct*"[Title/Abstract] OR "acute coronary syndrome"[Title/Abstract] OR "limb ischemia"[Title/Abstract] OR "limb ischaemia"[Title/Abstract] OR "peripheral embol*"[Title/Abstract] OR "renal infarct*"[Title/Abstract] OR "splenic infarct*"[Title/Abstract] OR "mesenteric ischemia"[Title/Abstract] OR "visceral infarct*"[Title/Abstract] OR "retinal artery occlusion"[Title/Abstract] OR "pulmonary embol*"[Title/Abstract]) | 1929 |
